# Evaluation of the expression of bone marrow‐derived mesenchymal stem cells and cancer‐associated fibroblasts in the stroma of gastric cancer tissue

**DOI:** 10.1002/ags3.12347

**Published:** 2020-05-27

**Authors:** Taizan Minami, Keishiro Aoyagi, Akihiko Kawahara, Naotaka Murakami, Taro Isobe, Yuya Tanaka, Hideaki Kaku, Fumihiko Fujita, Yoshito Akagi

**Affiliations:** ^1^ Department of Surgery Kurume University School of Medicine Fukuoka Japan; ^2^ Department of Diagnostic Pathology Kurume University School of Medicine Fukuoka Japan

**Keywords:** BM‐MSC, CAF, gastric cancer, immunohistochemistry

## Abstract

**Aim:**

Cancer‐associated fibroblasts (CAFs) generated by bone marrow‐derived mesenchymal stem cells (BM‐MSCs) play an important role in cancer progression. In this study, we investigated the relationships of BM‐MSCs and CAFs in resected gastric cancers with the clinicopathological factors of patients.

**Methods:**

We analyzed 120 gastric cancer patients who underwent gastrectomy. Immunostaining was performed with an anti‐CD271 antibody (BM‐MSCs) and anti‐α‐smooth muscle actin (αSMA) antibody (CAFs). Staining intensity was used to divide patients into low and high expression groups. Observation sites in cancer tissues were invasive, central, and whole portions.

**Results:**

Expression of αSMA was significantly related to depth of tumor invasion (T), lymph node metastasis (N), lymphatic invasion (ly), venous invasion (v), and stage. Expression of CD271 was significantly related to v, stage, stromal volume, and tumor infiltration pattern (INF). Overall survival (OS) of the high expression group was significantly lower than that of the low expression group for both αSMA and CD271. Multivariate analysis showed that N, αSMA (whole), and CD271 (invasive) were independent prognostic factors.

**Conclusions:**

Cancer‐associated fibroblasts and BM‐MSCs are related to the progression, invasion, and prognosis of gastric cancer and may be therapeutic targets of gastric cancer.

## Graphical abstract

We immunohistochemically examined bone marrow‐derived mesenchymal stem cells and cancer‐associated fibroblasts in gastric cancer tissues and obtained results indicating that they were associated with tumor infiltration and stroma, and closely related to prognosis.

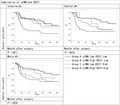

## 1 INTRODUCTION

Gastric cancer is a typical malignancy that occurs in the digestive tract. In worldwide cancer statistics, the number of gastric cancer cases ranks fifth, while the number of deaths ranks third.^1^ Prognosis for patients with advanced and recurrent gastric cancer is still poor, despite recent advances in chemotherapy and molecular targeted therapeutics with the desire for further identification of additional therapeutic targets.

Mesenchymal stem cells (MSCs) are somatic stem cells derived from mesodermal tissue (mesenchyma), which are present in all tissues. Among tissue stem cells, although MSCs are considered to differentiate into various mesenchymal cell types such as bone, cartilage, muscle, fat, and nerves, MSCs originally exist in bone marrow, and MSCs that are isolated from bone marrow can reconstitute various mesenchymal tissues.^2^

Changes in cancer cells themselves along with microenvironmental changes in the cancer surrounding the stroma are critically involved the development and metastasis of cancer. The peritumoral microenvironment includes various stromal cell types, cancer‐associated fibroblasts (CAFs), endothelial cells, adipocytes, and immune cells.^3^ Among these cell types, CAFs are related to tumor invasion and metastasis.^4^, ^5^ Furthermore, CAFs have recently been shown to be derived from MSCs^6^ with bone marrow‐derived MSCs (BM‐MSCs) mobilized via circulating blood being of particular interest. BM‐MSCs have the ability to differentiate into a wide variety of mesenchymal cell types and are involved in epithelial cell regeneration and angiogenesis, even during tissue repair. However, the impact and the role of BM‐MSCs in the tumor microenvironment are largely unknown.^7^ Although BM‐MSCs differentiate into CAFs, epithelial‐mesenchymal transition (EMT) and cancer stem cell (CSC) recovery have been reported to enhance the proliferation and invasion of gastric cancer cells in vitro and in vivo.^6^ However, there are few reports on BM‐MSCs in gastric cancer tissue. In this study, the relationships of BM‐MSCs and CAFs with the clinicopathological factors and prognosis of gastric cancer patients were examined immunohistochemically in resected gastric cancer tissues.

## 2 METHODS

### 2.1 Patients

This study was approved by the Institutional Review Board of Kurume University School of Medicine (ID: 19117) based on the Ethical Guidelines for Clinical Research of the Ministry of Health, Labour, and Welfare of Japan. Patients who underwent gastrectomy for gastric cancer at Kurume University Hospital agreed to donate surgically resected tumor specimens for research purposes. Written informed consent was obtained from all patients.

A total of 120 cases out of 706 gastric cancer surgery patients at Kurume University Hospital from January 2005 to December 2010 were chosen randomly. Subsequently, intramucosal cancer, residual gastric cancer, multiple cancers, and post‐ESD cases were excluded. Finally, 88 males and 32 females with a median age of 71.5 years (minimum age: 38; maximum age: 87) were enrolled in the study. The median observation period was 51 months with the shortest observation period as 5 months and longest observation period as 128 months.

### 2.2 Immunohistochemical staining

Immunohistochemical staining was performed using paraffin‐embedded sections, including the deepest part of the tumor in each case, with an anti‐CD271 (BM‐MSC marker) antibody^8^ and anti‐α‐smooth muscle actin (αSMA; CAF marker) antibody.^9^ Several researchers previously isolated MSCs from bone marrow using an anti‐CD271 antibody.^8^, ^10^, ^11^, ^12^ Therefore, CD271 is considered a marker for BM‐MSCs. CD271 is also known as affinity nerve growth factor receptor (LNGFR), nerve growth factor receptor (NGFR), and p75NTR (neurotrophin receptor), and belongs to the tumor necrosis factor superfamily.^13^

Paraffin‐embedded tissue samples were sectioned at 4 μm thicknesses, mounted on PLATINUM PRO‐coated slides (Matsunami Glass Ind., Ltd.), and labeled with antibodies using a Bond‐III autostainer (Leica Microsystems): Antibodies against CD271 (Abcam; diluted 1:200 in Dako REAL™ Antibody Diluent [Dako]) and αSMA (DakoCytomation; diluted 1:400 in Dako REAL™ Antibody Diluent) were used as primary antibodies. For CD271 staining, heat treatment was applied in Epitope Recovery Solution 2 (pH 9.0) for 30 minutes, followed by incubation with the anti‐CD271 antibody for 15 minutes. For αSMA staining, the antibody was applied without antigen recovery for 15 minutes. The automated system used for detection was a refined polymer detection system (Leica Microsystems) with horseradish peroxidase‐polymer as the secondary antibody, 3,3′‐Diaminobenzidine was used as the chromogen. The negative control for both αSMA and CD271 was phosphate‐buffered saline instead of primary antibody. The positive control for αSMA was the proper muscle layer of the stomach, and that for CD271 was melanoma tissue.

### 2.3 Evaluation of αSMA and CD271 expression

**FIGURE 1:**
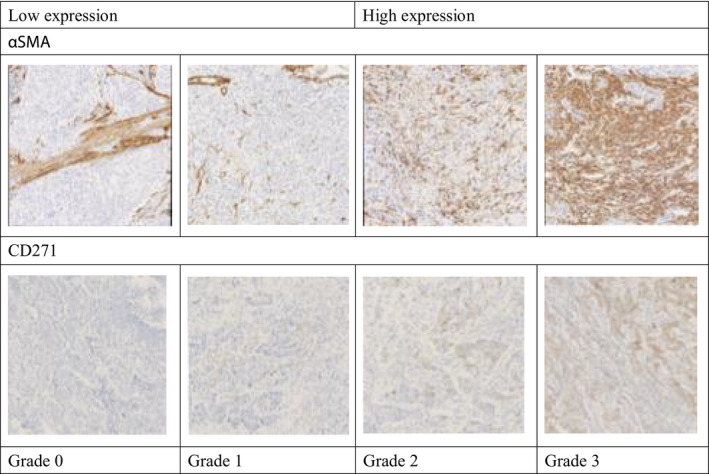
Grade 0: α‐smooth muscle actin (αSMA) expression was observed in vascular smooth muscle and muscularis propria, with no expression in myofibroblasts of cancer stroma. No cells were stained for CD271 protein. Grades 1‐3: αSMA expressed was detected in the fibroblast. CD 271 expression in the cytoplasm of cells was observed in the cancer stroma. The staining intensity for both αSMA and CD271 was scored as 0‐3. Scores 0 and 1 were defined as weak staining, while scores 3 and 4 were defined as strong staining. (Magnification for all histological image, ×100.)

To evaluate myofibroblasts as CAFs, αSMA was stained in myofibroblasts of the tumor stroma, vascular smooth muscles, proper muscle layer, and mucosal muscle plate. CD271 was stained in the cytoplasm of cells in the tumor stroma as BM‐MSCs. We counted positively stained cells in all fields of one section containing the deepest part of the tumor. We examined positive cases to determine the immunoreactivity for αSMA or CD271 in the cancer stroma, and evaluated the staining intensity under 200× magnification. The staining intensity was scored as 0‐3 to examine the association with clinicopathological factors (Figure 1). Furthermore, the staining intensity scores were divided into weak staining (0 and 1) and strong staining (2 and 3) to examine correlations with prognosis. The observation sites in the tumor were invasive, central, and whole portions. The samples were observed by two physicians for evaluation, and finally results were confirmed by a pathologist.

### 2.4 Statistical analysis

Statistical analysis was performed using JMP (version 2018). Statistical significance of analytical data was evaluated using the chi‐squared test or *t*‐test. *P* < .05 was deemed as statistically significant. The cumulative survival time was calculated using the Kaplan‐Meier method and analyzed by the log‐rank test. The stepwise method was used for multivariate analysis to calculate independent prognostic factors after prognostic variables were identified by univariate analysis. Spearman rank correlation coefficient analysis was used for comparisons of staining intensity between αSMA expression and CD271 expression in the invasive, central, and whole portions.

### 2.5 Terms

Clinicopathological terms were used in compliance with the Japanese classification of gastric carcinoma 3rd English edition.^14^

## 3 RESULTS

### 3.1 Expression of αSMA and CD271, and clinicopathological factors

α‐smooth muscle actin was associated with some clinicopathological factors (depth of tumor invasion (T): invasive, central, and whole (*P* = .003, .004, and .013, respectively); lymph node metastasis (N): central (*P* = .039); lymphatic invasion (ly): invasive and central (*P* = .023 and 0.019, respectively); venous invasion (v): center (*P* = .031); stage: central (*P* = .031)). However, no associations were found with age, sex, gross type, histology, stromal volume, or tumor infiltration pattern (INF) (Table 1). Furthermore, although CD271 was associated with some clinicopathological factors (v: central and whole [*P* = .021 and .035, respectively]; tissue type: central [*P* = .045]; stage: invasive [*P* = .042]; stromal volume: invasive [*P* = .024]; INF: central [*P* = .015]), no associations were found with age, sex, gross type, T factor, N factor, or ly (Table 2).

**TABLE 1:** Relationships between expression of α‐smooth muscle actin (αSMA) and clinicopathological factors

Factor	Patients	αSMA (invasive/central/whole)				*P* value (invasive/central/whole)
		0	1	2	3	
Age (y)						
<50	32	7/10/9	8/6/7	7/6/7	10/10/9	.95/.38/.44
≧50	88	16/15/13	26/20/25	19/24/22	27/29/28	
Gender						
Male	88	15/18/16	25/18/22	17/17/17	31/35/33	.28/.07/.12
Female	32	8/7/6	9/8/10	9/13/12	6/4/4	
T classification						
T1	28	10/10/8	8/7/10	2/3/2	8/8/8	.003/.004/.013
T2	37	8/6/6	13/11/14	12/14/11	4/6/6	
T3	51	4/9/8	12/8/7	11/10/14	24/24/22	
T4	4	1/0/0	1/0/1	1/3/2	1/1/1	
N classification						
N0	56	12/14/12	16/18/20	13/11/11	15/13/13	.142/.039/.101
N1	30	9/6/6	9/2/3	4/11/11	8/11/10	
N2	29	2/4/3	7/5/8	9/8/7	11/12/11	
N3	5	0/1/1	2/1/1	0/0/0	3/3/3	
ly classification						
ly0	12	6/5/5	2/3/3	1/1/1	3/3/3	.023/.019/.113
ly1	29	5/7/5	6/11/12	14/6/7	4/5/5	
ly2	50	6/6/6	19/10/12	4/12/11	21/22/21	
ly3	29	6/7/6	7/2/5	7/11/10	9/9/8	
V classification						
V0	39	10/11/9	9/13/15	13/7/7	7/8/8	.062/.031/.057
V1	65	10/9/9	19/11/13	9/17/16	27/28/27	
V2	16	3/5/4	6/2/4	4/6/6	3/3/2	
V3	0	0/0/0	0/0/0	0/0/0	0/0/0	
Tissue type						
tub	43	10/8/7	9/7/9	7/9/9	17/19/18	.266/.132/.241
pap	20	3/5/4	9/3/6	5/8/6	3/4/4	
por	28	2/3/2	7/7/8	7/6/7	12/12/11	
muc	5	1/2/2	2/1/1	1/2/2	1/0/0	
sig	24	7/7/7	7/8/8	6/5/5	4/4/4	
Stage						
I	45	12/13/11	15/15/18	10/9/8	8/8/8	.395/.031/.148
II	29	6/4/4	7/5/6	6/10/9	10/10/10	
III	26	2/3/3	7/4/5	5/6/6	12/13/12	
IV	20	3/5/4	5/2/3	5/5/6	7/8/7	
Stromal volume						
med	55	12/13/11	17/10/15	12/17/16	14/15/13	.575/.287/.343
int	29	4/3/3	8/6/6	4/6/6	13/14/14	
sci	36	7/9/8	9/10/11	10/7/7	10/10/10	
INF						
a	47	11/11/9	12/9/12	10/12/12	14/15/14	.894/.843/.956
b	44	7/6/6	10/12/12	8/12/11	14/15/14	
c	29	5/8/7	7/6/7	8/6/6	9/9/9	

Note

T1 tumor confined to the submucosa, T2 tumor invades the muscularis propria, T3 tumor invades the subserosa, T4 tumor invasion is contiguous to or exposed beyond the serosa or tumor invades adjacent structures, N0 no regional lymph node metastasis, N1 metastasis in one to two regional lymph nodes, N2 metastasis in three to six regional lymph nodes, N3 metastasis in seven or more regional lymph nodes, ly0 no lymphatic invasion, ly1 minimal lymphatic invasion, ly2 moderate lymphatic invasion, ly3 marked lymphatic invasion, v0 no venous invasion, v1 minimal venous invasion, v2 moderate venous invasion, v3 marked venous invasion, tub tubular adenocarcinoma, pap papillary adenocarcinoma, por poorly differentiated adenocarcinoma, muc mucinous adenocarcinoma, sig signet‐ring cell carcinoma, med sparse stroma, sci abundant stroma, int the quality of stroma is intermediate between med and sci, INFa tumor displays expanding growth with a distinct border from the surrounding tissue, INFb tumor shows an intermediate pattern between INFa and INFc, INFc tumor displays infiltrative growth with no distinct border with the surrounding tissue

**TABLE 2:** Relationships between expression of CD271 and clinicopathological factors

Factor	Patients	CD271 (invasive/central/whole)				*P* value (invasive/central/whole)
		0	1	2	3	
Age (y)						
<50	32	7/12/6	11/15/19	9/3/5	5/2/2	.943/.408/.400
≧50	88	23/48/21	26/14/37	24/17/21	15/9/9	
Gender						
Male	88	23/43/21	24/19/36	24/16/21	17/10/10	.387/.311/.143
Female	32	7/17/6	13/10/20	9/4/5	3/1/1	
T classification						
T1	28	8/13/6	11/7/14	5/5/5	4/3/3	.075/.364/.140
T2	37	13/24/13	13/8/17	6/3/5	5/2/2	
T3	51	7/21/6	12/12/23	21/12/16	11/6/6	
T4	4	2/2/2	1/2/2	1/0/0	0/0/0	
N classification						
N0	56	13/29/12	20/13/28	16/11/13	7/3/3	.09/.331/.276
N1	30	9/18/9	11/8/15	8/3/5	2/1/1	
N2	29	8/12/6	4/6/10	7/5/7	10/6/6	
N3	5	0/1/0	2/2/3	2/1/1	1/1/1	
ly classification						
ly0	12	4/7/4	3/1/4	3/3/3	2/1/1	.250/.250/.418
ly1	29	9/17/8	11/7/4	4/3/5	5/2/2	
ly2	50	12/22/10	13/15/24	20/11/14	5/2/2	
ly3	29	5/14/5	10/6/14	6/3/4	8/6/6	
V classification						
V0	39	14/23/12	14/9/18	8/6/8	3/1/1	.050/.021/.035
V1	65	12/28/11	19/19/32	23/13/17	11/5/5	
V2	16	4/9/4	4/1/6	2/1/1	6/5/5	
V3	0	0/0/0	0/0/0	0/0/0	0/0/0	
Tissue type						
tub	43	9/18/7	12/12/19	14/11/15	8/2/2	.463/.045/.115
pap	20	8/16/7	7/2/11	4/1/1	1/1/1	
por	28	6/15/6	9/4/13	8/4/4	5/5/5	
muc	5	3/3/3	0/2/1	1/0/1	1/0/0	
sig	24	4/8/4	9/9/12	6/4/5	5/3/3	
Stage						
I	45	12/24/11	19/11/23	7/6/7	7/4/4	.042/.102/.142
II	29	10/19/9	7/5/13	11/5/7	1/0/0	
III	26	5/10/4	7/9/13	9/5/7	5/2/2	
IV	20	3/7/3	4/4/7	6/4/5	7/5/5	
Stromal volume						
med	55	16/36/15	23/10/30	10/5/6	6/4/4	.024/.085/.071
int	29	7/10/5	3/8/9	11/8/12	8/3/3	
sci	36	7/14/7	11/11/17	12/7/8	6/4/4	
INF						
a	47	15/32/14	16/5/22	10/5/6	6/5/5	.396/.015/.280
b	44	9/17/7	15/16/22	11/9/13	9/2/2	
c	29	6/11/6	6/8/12	12/6/7	5/4/4	

Note

T1 tumor confined to the submucosa, T2 tumor invades the muscularis propria, T3 tumor invades the subserosa, T4 tumor invasion is contiguous to or exposed beyond the serosa or tumor invades adjacent structures, N0 no regional lymph node metastasis, N1 metastasis in one to two regional lymph nodes, N2 metastasis in three to six regional lymph nodes, N3 metastasis in seven or more regional lymph nodes, ly0 no lymphatic invasion, ly1 minimal lymphatic invasion, ly2 moderate lymphatic invasion, ly3 marked lymphatic invasion, v0 no venous invasion, v1 minimal venous invasion, v2 moderate venous invasion, v3 marked venous invasion, tub tubular adenocarcinoma, pap papillary adenocarcinoma, por poorly differentiated adenocarcinoma, muc mucinous adenocarcinoma, sig signet‐ring cell carcinoma, med sparse stroma, sci abundant stroma, int the quality of stroma is intermediate between med and sci, INFa tumor displays expanding growth with a distinct border from the surrounding tissue, INFb tumor shows an intermediate pattern between INFa and INFc, INFc tumor displays infiltrative growth with no distinct border with the surrounding tissue.

### 3.2 Correlations of αSMA and CD271 expression

Expression of αSMA and CD271 tended to be correlated at the invasive, central, and whole portions (*P* = .08, .07, and .08, respectively; Table 3).

**TABLE 3:** Correlations of α‐smooth muscle actin (αSMA) and CD271 expression

	CD271 Low n (%)	CD271 High n (%)
α SMA low	Group A	Group B
	Invasive 42 (35.0)	14 (11.6)
	Central 47 (39.1)	5 (4.1)
	Whole 47 (39.1)	8 (6.67)
α SMA high	Group C	Group D
	26 (21.6)	38 (31.6)
	42 (35.0)	26 (21.6)
	36 (30.0)	29 (24.1)

Note

Groups A‐D are αSMA Low and CD271 Low, αSMA Low and CD271 High, αSMA High and CD271 Low, and αSMA High and CD271 High, respectively (invasive: *P* = .08; central: *P* = .07; whole: *P* = .08).

By Spearman rank correlation coefficient analysis, there were correlation between αSMA expression and CD271 expression in the invasive portion (*r* = .3922, *P* < .0001), and whole portion (*r* = .3450, *P* < .0001), and strong correlation in the central portion (*r* = .4225, *P* < .0001).

### 3.3 Relationship of prognosis with the expression of αSMA and CD271

The 3‐ and 5‐year survival rates in the αSMA high expression group were 47.5%/37.8%, 46.4%/35.2%, and 43.7%/33.7% in terms of the invasive, central, and whole portions, respectively, compared with 76.7%/53.7%, 79.9%/57.8%, and 80.8%/58.2%, respectively in the αSMA low expression group. The 3‐ and 5‐year survival rates in the CD271 high expression group were 46.7%/31.7%, 40.0%/31.5%, and 44.5%/33.3% in terms of the invasive, central, and whole portions, respectively, compared with 74.3%/54.2%, 68.4%/49.4%, and 68.5%/49.6%, respectively in the CD271 low expression group.

**FIGURE 2:**
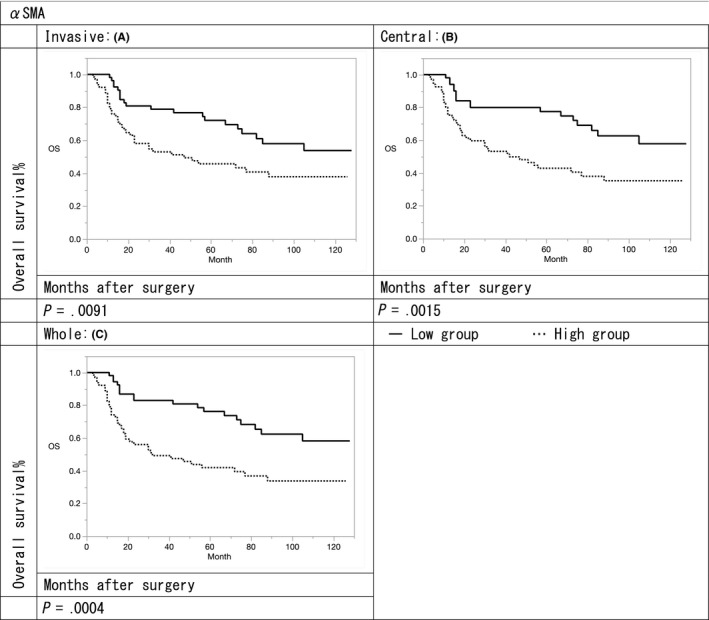
Relationship of prognosis with the expression of α‐smooth muscle actin (SMA): (A) survival curves of α‐SMA in invasive portion (B) survival curves of αSMA in central portion (C) survival curves of α‐SMA in whole portion

**FIGURE 3:**
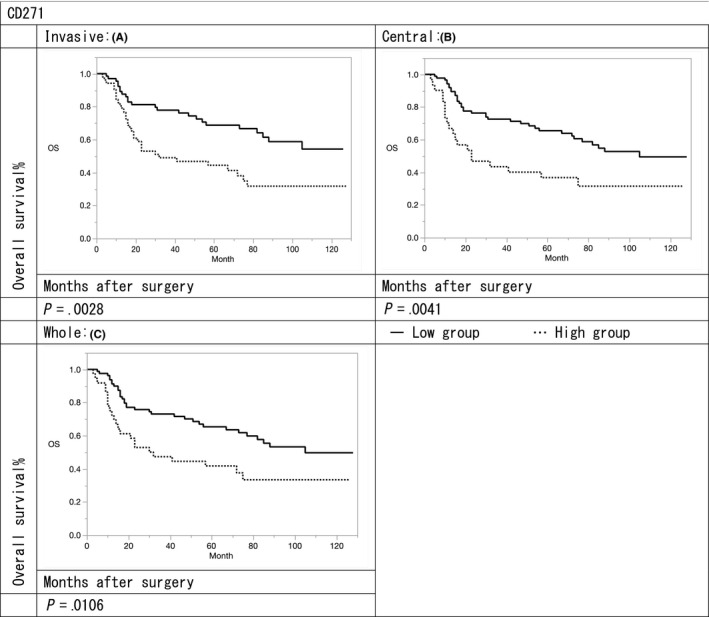
Relationship of prognosis with the expression of CD271: (A) survival curves of CD271 in invasive portion (B) survival curves of CD271 in central portion (C) survival curves of CD271 in whole portion

**FIGURE 4:**
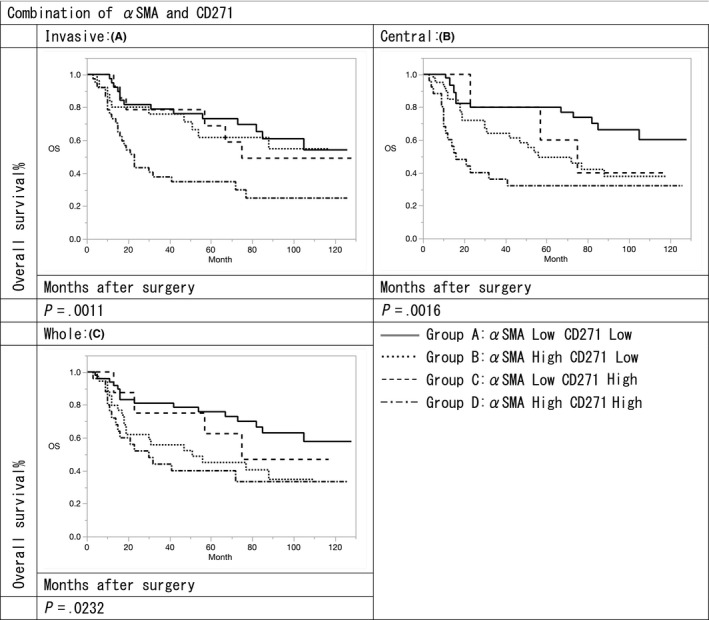
Groups A‐D are α‐smooth muscle actin (αSMA) Low and CD271 Low, αSMA Low and CD271 High, αSMA High and CD271 Low, and αSMA High and CD271 High, respectively. Overall survival of both αSMA and CD271 high expression groups was significantly poorer than that of the both αSMA and CD271 low expression groups in terms of invasive, central, and whole portions (*P* = .0011, .016, and .0232, respectively)

The overall survival (OS) rates of αSMA and CD271 high expression groups were significantly lower to those of the low expression groups in terms of invasive, central, and whole portions (αSMA: *P* = .009, .001, and .0004; CD271: *P* = .002, .004, and .010, respectively) (Figures 2A‐C and 3A‐C). Furthermore, the median survival times for both αSMA and CD271 low expression groups were 42, 47, and 50 months in terms of invasive, central, and whole portions, respectively. Median survival times of αSMA low expression and CD271 high expression groups were 26, 42, and 37 months in terms of invasive, central, and whole portions, whereas those of αSMA high expression and CD271 low expression groups were 38, 26, and 25 months, respectively. Median survival times of both αSMA and CD271 high expression groups were 14, 5, and 8 months in terms of invasive, central, and whole portions, respectively. The OS of both αSMA and CD271 high expression groups was significantly poorer in terms of invasive, central, and whole portions compared with both low expression groups (*P* = .001, .001, and .023, respectively) (Figure 4A‐C).

### 3.4 Multivariate analysis

As factors involved in the prognosis of gastric cancer, according to univariate analysis, T factor (*P* = .0001), N factor (*P* = .0001), v (*P* = .0023), αSMA expression in the invasive, central, and whole portions, and CD271 expression in invasive, central, and whole portions (*P* = .0028, .0041, and .0106, respectively) were significant prognostic factors. In the multivariate analysis, N factor (HR: 3.170, 95% CI: 1.779‐5.920; *P* < .0001), αSMA expression in the whole portion (HR: 1.902, 95% CI: 1.078‐3.478; *P* = .0260), and CD271 expression in the invasive portion (HR: 2.080, 95% CI: 1.206‐3.636; *P* = .0084) were independent prognostic factors (Table 4).

**TABLE 4:** Univariate and multivariate analyses of prognostic factors of survival

Factors	Univariate *P* value	Multivariate(stepwise)		
		HR	95% CI	*P* value
Age <65 vs ≧65	.522			
Sex	.321			
T classification T1‐2 vs T3‐4	.0001	0.881	0.511‐1.519	.2439
N classification N0 vs N1‐3	.0001	3.170	1.779‐5.920	<.0001
V classification V0 vs V1‐3	.0023	0.576	0.304‐1.092	.1878
Stroma sci vs int + med	.2715			
Tissue type	.5038			
α‐smooth muscle actin expression				
Invasive	.0091	0.659	0.399‐1.823	.0923
Central	.0015	0.559	0.329‐0.994	.0767
Whole	.0004	1.902	1.078‐3.478	.0260
CD271 expression				
Invasive	.0028	2.080	1.206‐3.636	.0084
Central	.0041	0.493	0.291‐1.091	.0745
Whole	.0106	0.698	0.459‐1.022	.1545

Note

T1 tumor confined to the submucosa, T2 tumor invades the muscularis propria, T3 tumor invades the subserosa, T4 tumor invasion is contiguous to or exposed beyond the serosa or tumor invades adjacent structures, N0 no regional lymph node metastasis, N1 metastasis in one to two regional lymph nodes, N2 metastasis in three to six regional lymph nodes, N3 metastasis in seven or more regional lymph nodes, v0 no venous invasion, v1 minimal venous invasion, v2 moderate venous invasion, v3 marked venous invasion, med sparse stroma, sci abundant stroma, int the quality of stroma is intermediate between med and sci, HR hazard ratio, CI confidence interval.

Therefore, the presence of CAFs in the whole tumor and BM‐MSCs in the invasive portion was considered to be involved in the prognosis of gastric cancer.

## 4 DISCUSSION

In this study, gastric cancer tissues were immunostained for αSMA and CD271 to examine CAFs and BM‐MSCs, respectively, with respect to gastric cancer stroma. CAFs were correlated with the T factor, Ly factor, V factor, N and stage, whereas BM‐MSCs were correlated with the V factor, stromal volume and INF. These observations suggested that αSMA was involved in tumor invasion and metastasis of CAFs because of its relationships with T and N, while CD271 was involved in the tumor stroma of BM‐MSCs because of its relationships with stromal volume and INF. Furthermore, BM‐MSCs in the invasive portion of the tumor and CAFs in the tumor stroma were poor independent prognostic factors.

As tumors grow larger, they need to obtain oxygen and nutrition from newly developed vessels. CAFs generated from BM‐MSCs were recruited to the tumor after first accumulating in the peripheral blood.^15^ Histologically, many BM‐MSCs were observed around cancer cells in the invasive portion of the tumor, together with CAFs throughout the tumor stroma.

Epithelial‐mesenchymal transition was originally reported by Hay et al as a phenomenon observed early in ontogeny.^16^ The importance of EMT for tumor growth progression has been reported in recent years. Cytokines that induce EMT include Wnt and TGF‐β produced by cancer cells.^17^ Furthermore, TGF‐β has been reported to effectively enhance EMT in cooperation with TNF‐α and other growth factors.^18^, ^19^, ^20^ The tumor microenvironment includes immune cells, CAFs, vascular tissues or lymph tissues, and MSCs. These cells engage in cross‐talk with tumor cells and secrete matrix metalloproteinases that promote the proliferation and invasion of tumor cells together with chemokines and growth factors.^21^, ^22^ Although tumorigenesis is widely known to be regulated by interactions between tumor cells and CAFs, the exact origin and functions of CAFs are unknown. Kabashima‐Niibe et al^23^ focused on EMT regulation of pancreatic cancer cells and the role of CAFs in cancer progression and showed that CAF‐induced EMT is involved in the invasion and metastasis of pancreatic cancer. Furthermore, in an in vitro coculture experiment using a double chamber, Fujiwaraet al^24^ confirmed that the proportion of CD271‐positive pancreatic stellate cells transiently increases in cocultures with pancreatic cancer cells, and then subsequently decreases, suggesting that it plays a role in the development of pancreatic cancer. Recently, several studies showed that MSCs play a role in tumor promotion promote. Maeterns et al^25^ reported that co‐injection of BM‐MSCs and tumor cells into mice increased intratumor lymphatic vessel density and tumor growth. Zhang et al^26^ reported that expression of vascular endothelial growth factor receptor and prospero homeobox protein 1 was increased in SGC‐7901 and HGC‐27 cells treated with BM‐MSC condition medium. However, we believe that our study is first to determine the prognosis of human gastric cancer related to expression of BM‐MSCs.

Bone marrow‐MSCs are induced in tumor tissue by IL‐6, Wnt‐5a, and BMP4 expressed in the tumor tissue with further differentiation to CAFs promoted by TGF‐β1 and SDF‐1α.^27^

Senba^28^ performed cocultures by direct contact with MKN‐7 cells derived from differentiated gastric adenocarcinoma and UE6E7T‐12 cells derived from BM‐MSCs, and indicated that BM‐MSCs induce EMT of gastric cancer cells and increase the number of CSCs by showing increased expression of EMT transcription factor vimentin, Snail, and CD133 that are markers of CSCs. Moreover, in vivo studies have shown that the size of tumors after subcutaneous co‐injection of MKN‐7 and UE6E7T‐12 cells into mice is significantly larger than that of tumors after injection of MKN‐7 cells alone. This finding suggested that BM‐MSCs contributed to tumor formation as CAFs by observing an increase in fibroblasts derived from UE6E7T‐12 cells after subcutaneous co‐injection of MKN‐7 and UE6E7T‐12 cells.

Moreover, it has been reported that the direct contact of BM‐MSCs with cancer cells induces EMT and stem cell recovery of cancer cells via the paracrine actions of TGF‐β derived from BM‐MSCs, along with the autocrine action of Wnt‐5a secreted from cancer cells.^28^

In this study, we immunohistochemically examined BM‐MSCs and CAFs in gastric cancer tissues and obtained results indicating that they were associated with tumor infiltration and stroma, and closely related to prognosis. In the future, we will investigate the relationship of BM‐MSCs and CAFs with EMT, CSC recovery, and expression of related cytokines to identify new biomarkers and therapeutic targets for gastric cancer. Recently, MSCs have been considered ideal gene career for therapy of malignant disease, because they are safe and can migrate tumors.^29^, ^30^ So, MSCs are considered a potential gene delivery system for gastric cancer. Moreover, immune check point inhibitors were recently approved for therapy of gastric cancer. Luz‐Crowford et al^31^ reported that MSCs had an immunosuppressive effect on Th17 cells through upregulated of PD‐L1 expression. Clarification of the relationship between MSCs and immune responses is considered a very interesting issue for future studies.

## DISCLOSURE

Funding: This study was not funded.

Conflicts of interest: The authors declare no conflicts of interests for this article.

Author Contribution: The conception or design of the work, or acquisition, analysis or interpretation of data for the work: Taizan Minami, Keishiro Aoyagi, Taro Isobe, Yuya Tanaka, Hideaki Kaku. Drafting the work or revising it critically for important intellectual content: Taizan Minami, Keishiro Aoyagi. Analysis of histological findings or performing immunohistochemical staining: Taizan Minami, Keishiro Aoyagi, Akihiko Kawahara. Final approval of the version to be published; Keishiro Aoyagi, Naotaka Murakami, Fumihiko Fujita, Yoshito Akagi. Agreement to be accountable for all aspects of the work in ensuring that questions related to the accuracy or integrity of any part of the work: Yoshito Akagi.

## ETHICS APPROVAL

All procedures performed in studies involving human participants were in accordance with the ethics standards of the institutional and/or national research committee and with the 1964 Helsinki Declaration and its later amendments or comparable ethical standards.

## INFORMED CONSENT

Informed consent was obtained from all individual participants included in this study.
